# Forage Turnip (*Brassica rapa* L.) as a Dietary Supplement to Improve Meat Quality

**DOI:** 10.3390/ani15091277

**Published:** 2025-04-30

**Authors:** Romina Rodríguez-Pereira, Ignacio Subiabre, Cristian J. Moscoso, Carolina E. Realini, Rodrigo Morales

**Affiliations:** 1 Escuela de Medicina Veterinaria, Facultad de Recursos Naturales y Medicina Veterinaria, Universidad Santo Tomás, Talca 3460000, Chile; 2 Instituto de Investigaciones Agropecuarias, INIA Remehue, Ruta 5 Km 8, Osorno 5290000, Chile; 3 AgResearch Limited, Te Ohu Rangahau Kai, Massey University Campus, University Ave., Palmerston North 4474, New Zealand; 4 Instituto de Investigaciones Agropecuarias, INIA Ururi, Magallanes 1865, Arica 1000000, Chile

**Keywords:** beef quality, *Brassica rapa subsp. rapa*, fatty acids, summer supplementation, Chile

## Abstract

Summer turnips (*Brassica rapa* L.) have become one of the main supplementary crops in ruminant livestock systems in southern Chile because of accelerated forage growth as well as greater forage yield and nutritive value in the dry season. This study investigated the effects of forage turnip supplementation on the physicochemical and sensory quality of beef from steers. Twenty-seven Holstein–Friesian steers were allocated to one of three dietary treatments: pasture plus concentrate (Control), 50% turnip with a basal diet of pasture hay and rolled corn (T50), and 70% turnip with the same basal diet (T70). Carcass yields and physicochemical and sensory beef attributes, including fatty acid composition of intramuscular fat (IMF) in lean tissue, were measured. Compared to the Control diet, finishing steers on 50% or 70% turnips increased meat redness (a* > 25.9 vs. 22.9 in Control), IMF (1.79% in T50 vs. 1.12% in Control), polyunsaturated fatty acids (PUFAs) (especially n-3), cholesterol, and specific minerals (sodium, manganese and iron); this resulted in a reduction in the subcutaneous fat thickness (0.29 cm in T50, 0.25 cm in T70 vs. 0.51 cm in Control) and shear force of cooked meat (*p* < 0.05). However, no differences were found between diets in beef juiciness, flavor, and tenderness assessed by trained panelists (*p* > 0.05). Increasing forage turnip inclusion to 70% resulted in similar beef quality to the 50% inclusion level. Foraged turnips present a promising strategy for producing high-quality beef during periods of limited pasture growth.

## Simple Summary

The inclusion of summer turnips (*Brassica rapa* L.) into ruminant livestock diets in southern Chile offers significant benefits, particularly during the dry season when forage quality is compromised. This research, examining the effects of incorporating turnip into the diet of Holstein–Friesian steers, highlights significant enhancements in the physicochemical characteristics of beef. These improvements include a boost in the redness of the meat, a rise in intramuscular fat, and an increase in polyunsaturated fatty acids, particularly *n*-3 fatty acids. These enhancements meet consumer demand for healthier and more nutritious beef. Additionally, turnip supplementation reduces subcutaneous fat thickness and shear force, potentially improving carcass yield and tenderness without compromising sensory attributes like juiciness and flavor. This study shows that both 50% and 70% turnip inclusion levels maintain beef quality, offering flexibility in dietary formulations. This strategy not only addresses forage scarcity but also enhances the long-term sustainability and marketability of beef production, providing a valuable solution for livestock systems facing seasonal challenges.

## 1. Introduction

Consumers have shown increased interest in how meat is produced and the implications regarding animal welfare, the environment, and human health [1]. More natural and low-impact production systems, such as meat produced from grazing animals, have shown improvements in animal welfare and the environment as well as the nutritional profile compared with meat produced in intensive high-impact systems [1,2]. Grass-fed systems produce beef with less intramuscular fat (IMF) (2–3%), a better *n*-6:*n*-3 fatty acids ratio, and a higher concentration of conjugated linoleic acid (CLA) and *trans* vaccenic acid (C18:1 t11) compared to beef from grain-fed systems [3,4,5]. Nevertheless, beef produced in grazing conditions is being affected by global average temperature increase, which has significant implications for forage production and animal performance. Beef-finishing grazing systems in humid temperate regions are found in a few countries worldwide [6], with a broad range of beef production per hectare [7]. In these systems, animal feeding depends on pasture and is mostly managed under rainfed conditions. Pasture seasonal production distribution varies amply throughout the year [8], with low growth rates during winter and in dry summers [9]. In addition, pasture nutritional quality often limits production during the summer [10]. However, the scarcity of rainfall has caused forage growth rates in these pastures to decrease drastically during the summer period, severely limiting animal production during this period. This situation will be accentuated in drought years and even more so due to the effects of climate change; under present scenarios, temperature increases up to 4 °C and a reduction in the monthly accumulated rainfall are predicted for central-southern Chile (37–42 °S) by the year 2100 [11]. Therefore, these changes are expected to impact pasture growth rates negatively [12].

In this context, grass and crop species adapted to new climate scenarios are essential for the success of livestock production. A low-cost alternative for summer supplementation is forage crops, and fodder turnip (*Brassica rapa* L.) has raised special interest due to its rapid growth during spring, high dry matter (DM) yield, and high forage production (leaves and roots) of elevated nutritional quality, even during dry summers [13,14,15]. In this respect, turnip is a profitable supplementary crop that allows the extension of the pasture season and maintains the supply of nutrients and energy required by grazing animals to reach market target weights and consistent meat quality [15,16].

Studies on forage turnip have been conducted, assessing production performance and meat quality attributes in lambs and dairy cows. The effect of forage turnip on milk characteristics has been researched [17,18,19], as well as its effect on the sensory attributes of lamb meat [16,20,21]. However, studies evaluating the effect of forage turnip inclusion on beef cattle production parameters are limited, especially on meat quality attributes. Thus, we hypothesize that the dietary inclusion of turnips will allow for the maintenance of a forage diet in the summer and improve carcass yield and beef quality in grass-fed beef animals. This study aimed to evaluate the impact of forage turnip supplementation on the physicochemical and sensory quality of beef from Holstein–Friesian steers, particularly in climate change scenarios where summer pasture availability and quality are limited.

## 2. Materials and Methods

Animal procedures were reviewed and approved by the Instituto de Investigaciones Agropecuarias (INIA) Animal Ethics Committee according to the Animal Welfare Act 1999.

### 2.1. Experimental Location

This study was carried out at Instituto de Investigaciones Agropecuarias (INIA) Remehue (latitude 40°31′15.3” S; 73°03′55.8” W; altitude 35 m.a.s.l.; annual rainfall 1250 mm), in Osorno, Los Lagos Region, Chile, for 77 days from 12 January to 29 March (summer) following a 15-day adaptation period. During this adaptation phase, a gradual increase of grain and/or turnips was implemented to reach the target inclusion level according to each dietary treatment.

### 2.2. Animals and Diets

Twenty-seven Holstein–Friesian steers, born in spring, were chosen at 14 months old from the INIA-Remehue animal production unit. The experiment was conducted using a randomized complete block design. The steers, with an initial average body weight of 390.2 ± 14.3 kg, were evenly distributed based on their live body weight into one of three experimental treatments. Each treatment had three groups, with three steers in each, resulting in nine steers per treatment (*n* = 9 steers/treatment). For each treatment, three separate paddocks were designated using electric fencing. To ensure diets with equal energy content, concentrate, along with pasture hay and rolled corn, were utilized for both the Control and turnip-based diets, respectively. The pasture hay was harvested in the spring from the same paddock that the Control group used. The commercial concentrate (Concentrados Cisternas^®^, Osorno, Chile) was composed of the following ingredients (in g/kg, as-fed basis): wheat bran, 500; sifted oats, 250; oat ground beans, 60; rice bran, 50; marigold seeds, 50; peanuts, 50; and flax expeller, 40. The treatments involved three different diets for the steers. The Control diet consists of pasture and commercial concentrate. The second group (T50) and the third one (T70) received the same basal diet of hay and rolled corn (Table 1). Each diet was supplemented with a daily intake of 100 g per animal of mineral salts (Vetersal grazing, Santiago, Chile), which was provided in a loose form and manually mixed with the supplementary feeds. The nutritional composition of these diet components is detailed in Table 1. The turnip variety Rival (*Brassica rapa*) was sown in October, which is springtime, across two paddocks, measuring 0.7 and 1.2 hectares. Additional feed was made available in troughs, with each animal allocated 60 cm of linear feeder space. Water was provided freely using drinking troughs, and feed was distributed once daily. The amount of feed given was determined based on the goal of achieving a daily weight gain of 1 kg of live body weight, as outlined by AFRC [22].

### 2.3. Herbage Measurements

The pasture used in this study was an improved natural pasture using management strategies for increased yield and quality. Grazing management consisted of allowing variable grazing stripes to cattle based on the weekly offer of DM using electric fences (S20, Gallagher^®^, Hamilton, New Zealand). The pre-grazing sward height was recorded daily using a rising plate meter (F200, Farmworks^®^, Feilding, New Zealand) at random spots within the section scheduled for grazing the following day. For the estimation of the available DM of the pasture, the plate meter was calibrated every seven days according to Canseco et al. [23]. Post-grazing sward heights were recorded daily at random locations within each treatment area grazed the previous day. The mean herbage DMI per animal was estimated daily according to the difference between pre- and post-grazing herbage mass to ground level, divided by the number of steers per treatment and repetition. The dry matter of the forage turnip was estimated as the average of 10 measurements per paddock using a 0.5 m^2^ frame. Turnips were pulled out, and leaves were separated from the root to be weighed. The botanical composition of both the pasture and the forage crop was determined as the average of four measurements per replicate using a 0.5 m^2^ frame. The pasture was cut to ground level, and species were separated and dried in a forced air oven at 100 °C for 8 h. The samples were weighed to determine the percentage of each species and were expressed on a DM basis.

The botanical composition of the forages used in each dietary treatment is presented in Table 2. The Control group grazed a diverse temperate pasture, mainly composed of perennial ryegrass, with contributions from other grasses, legumes, and a moderate amount of dead material. In contrast, the T50 and T70 groups received forage based primarily on turnip crops. During the early part of this study, the turnip sward was highly uniform, but as the season progressed, the presence of weeds and dead material increased while the proportion of turnip declined.

Feed samples were collected at the beginning, middle, and end of the trial to determine DM, crude protein (CP), ash, and ether extract [24]. Metabolizable energy (ME) and neutral detergent fiber (NDF) were determined according to Sadzawka et al. [25], and soluble carbohydrates were determined according to MAFF [26].

### 2.4. Animal Measurements

Steers were weighed individually at the beginning of this study and every 15 days thereafter using a mechanical scale located approximately 1.0 km from the experimental paddocks. To minimize stress-related variability, all animals were handled uniformly, and weighing was conducted under consistent conditions, including a rest period prior to measurement. These data were used to estimate the evolution of live BW as well as partial and mean average daily gain (ADG).

### 2.5. Carcass Measurements

Steers were slaughtered at a commercial meat processing plant (FRIGOSORNO, Osorno, Chile) when animals reached the minimum market requirement of grade 1 for fat cover (440.1 ± 15.7 kg live weight). Steers from the Control group were slaughtered 21 days earlier than those in the turnip treatment groups. Steers were transported approximately 15 km from the experimental site to a commercial meat processing plant licensed for export. All animals were slaughtered on the same day following standard procedures. Briefly, animals were stunned using a captive bolt gun, followed by exsanguination. Electrical stimulation was applied for 30 s. Carcasses were suspended from the Achilles tendon, eviscerated, dressed, and then chilled at 0 °C. These procedures were conducted in accordance with commercial meat processing protocols to ensure consistency in carcass handling and minimize stress-related effects on meat quality. Hot carcass weights were recorded, and carcasses were chilled for 24 h at 4 ± 2 °C. Carcasses were split between the ninth and tenth rib to evaluate subcutaneous fat depth and pH. Three consecutive pH measurements were recorded by inserting the pH electrode from a portable calibrated pH meter with automatic temperature compensation (Hanna 99,163; Hanna Instruments, Woonsocket, RI, USA) in the *Longissimus thoracis et lumborum* (LTL) muscle. Dorsal fat thickness was measured with Vernier on the standardized AUS-MEAT site in LTL [27]. A section of the LTL muscle was removed from the ninth thoracic vertebra to the last lumbar vertebra of each carcass. This section was divided into three equal parts that were vacuum sealed and aged for 21 days at 4 ± 2 °C. The cranial section of the LTL muscle was used for sensory analysis, the center section for instrumental analysis, and the caudal part was frozen at −18 ± 2 °C until subsequent chemical analyses, proximal composition, and fatty acid profile evaluation. All analyses were carried out at the food quality laboratory of INIA Remehue (Osorno, Chile).

### 2.6. Meat Quality Measurements

Chemical proximate analysis (DM, protein, and ash) was measured according to the procedures described by AOAC [24], and mineral content was measured according to Sadzawka et al. [25]. All external fat was removed from the samples to determine intramuscular fat (IMF) by Soxhlet procedure using petroleum ether as solvent [24]. Instrumental meat color was measured using a Konica Minolta colorimeter (CR-400; Minolta Inc., Osaka, Japan) after 30 min of bloom time at room temperature. The L*, a*, and b* values were measured using Illuminant D65, an 8 mm diameter aperture, and a 2° standard observer. Subsequently, meat samples were cooked in an electric oven (EKA.KF 620 model, Famava, Santiago, Chile) pre-heated at 170 °C to an internal meat temperature of 71 °C. Meat samples were allowed to cool at room temperature and were subsequently kept for 24 h at 4 ± 2 °C. Using a metal hole punch, 9 cores of 1.27 cm in diameter were removed from each sample parallel to the orientation of the muscle fibers. Each muscle core was sheared perpendicular to the muscle fiber direction using a texturometer (TA-XT2i texture analysis, Stable Micro Systems Ltd., Godalming, UK) fitted with a Warner–Bratzler blade. The average shear force of the nine cores was used per sample and expressed as Newtons (Ns).

### 2.7. Fatty Acids Composition and Cholesterol Analyses

Animal feed samples were analyzed according to the methodology of direct transesterification and purification through thin layer chromatography described by Alves et al. [28]. Lipid extraction and methylation from the meat samples (1 g of lyophilized meat) were carried out according to Aldai et al. [29]. For quantitative purposes, 1 mL of internal standard (1 mg/mL of 23:0 methyl ester, n-23-M from Nu-Chek Prep Inc., Elysian, MN, USA) was added before the methylation. The content of methyl ester of fatty acids (FAMEs) was expressed in mg/100 g of fresh meat. The content of methyl ester of fatty acid was analyzed using gas chromatography equipment (GC) with a flow ionization detector GC-2010 Plus (Shimadzu^®^, Kyoto, Japan). A 100 m SP560 column (Supelco, Bellefonte, PA, USA) was used, operating in two temperature programs, one at 175 °C and another at 150 °C [30]. In addition, a 100 m SLB-IL111 ionic liquid column (Supelco, Bellefonte, PA, USA) [31] was used to confirm the identification of several biohydrogenation intermediates, such as CLA isomers. In both columns, hydrogen was used as carrier gas at a constant flow of 1 mL/min, injector and detector temperatures were set at 250 °C. For peak identification, the reference standards #463 and #603 were used, along with individual FAMEs (21:0, 23:0, 26:0), and a mixture of CLA #UC-59 M (9c, 11t-/8t, 10c-/11c, 13t-/10t, 12c-/8c, 10c-/9c, 11c-/10c, 12c-/11c, 13c-/11t, 13t-/10t, 12t-/9t, 11t-/8t, 10t-18:2), all of which were obtained from Nu-Chek Prep Inc. (Elysian, MN, USA). A mixture of isomers of linoleic acid (18:2 *n*-6) and linolenic acid (18:3 *n*-3) was acquired from Sigma-Aldrich (St. Louis, MO, USA) (#47791 y #47792, respectively; Supelco, Bellefonte, PA, USA). The branched-chain fatty acids (BCFAs) were identified using a mixture of bacterial FAME isomers obtained from Matreya (Pleasant Gap, PA, USA). Other non-conjugated dienes not included in the standard mixture were identified by their retention times and order of elution as reported in the literature and were confirmed using fractions of FAMEs obtained from Ag^+^-SPE cartridges [32]. For cholesterol, fat extraction was conducted according to the procedure described by Aldai et al. [29]. To the extracted fat, a basic NaOH methylation method was conducted in a methanolic base at 0.5N [33]. For quantitative purposes, an external standard cholesterol aliquot was added. Cholesterol content was expressed in mg/100 g of fresh meat and as a percentage (%) of the total FAMEs quantified. It was analyzed through high-performance liquid chromatography (HPLC) (CO- 2065 Plus Jasco^®^, Tokyo, Japan) using a Zorbax RX-SIL 4.6 × 250 mm column with a diode detector array (MD-2010 Plus; Jasco^®^, Tokyo, Japan) in a temperature program of HPLC at 30 °C. In the column, Isopropanol-hexane at 3% was used as a mobile phase at a constant flow of 0.8 mL/min^−1^.

### 2.8. Sensory Analysis

A panel of 11 trained members (six men and five women) participated in the sensory analysis. Training and testing sessions were conducted at the Sensory Analysis Laboratory at INIA Remehue (Osorno, Chile). Panelists were selected from 30 initial volunteers without previous experience in sensory evaluation, and training was carried out in accordance with the American Society for Testing and Materials [34] and International Organization for Standardization (ISO) [35]. The panel has over 8 years of experience in beef sensory evaluation. The sensory laboratory was designed according to ISO standards, with individual booths, and samples were evaluated in a sequence to avoid the effect of the order of presentation of samples and the effect of first order or delay [36]. Panelists in each session first evaluated meat and fat color intensity and marbling level in three raw samples. This was followed by the evaluation of flavor, tenderness, and juiciness of cooked meat samples. Panelists evaluated samples in duplicate, analyzing three different samples per session, completing a total of 18 sessions. The descriptors were quantified using a hybrid scale ranging from 0 (absence) to 10 (maximum intensity) [37].

### 2.9. Statistical Analysis

Dry matter intake data were analyzed by ANOVA, including the type of finishing (Control, T50, and T70) as a fixed effect, and the paddock (three paddocks per treatment) was included as a random term in the model. The paddock was the experimental unit, and steers were the sampling unit. There were nine paddocks in total, each treatment had three paddocks. Each paddock was assigned three animals, and it was balanced for weight for each treatment. Carcass traits were analyzed by ANCOVA, including final live BW as a covariate, treatment as a fixed effect, and paddock as a complete random effect. Meat quality characteristics (shear force and meat and fat color) were analyzed by ANOVA, including treatment as a fixed effect, and paddock as a complete random effect. The fatty acid profile of meat was analyzed by ANCOVA, including FAMEs total content as a covariate, treatment as a fixed effect, and paddock as a complete random effect. Sensory analysis data were analyzed by ANOVA, including treatment, panelists, and repetition as fixed effects, and sessions and paddock as random effects. The Tukey test was used to detect differences among means (*p* < 0.05). All data were analyzed using mixed models, and the analysis was carried out with the statistical software XLSTAT version 2017.1

## 3. Results and Discussion

### 3.1. Animal Performance and Carcass Traits

The initial and final weight and carcass characteristics of steers are shown in Table 3. The initial weight did not differ between treatments (*p* = 0.936). Differences were observed in DMI (*p* < 0.05), with values of 8.69 kg/d for the Control group, 9.29 kg/d for T50, and 9.76 kg/d for T70. However, no differences (*p* > 0.05) were found between the turnip diets. Significant differences in final live weight and Average Daily Gain (ADG) were observed (*p* < 0.05), with the Control group showing higher values compared to the T70 group. Despite the increased DMI observed in the T70 group, this did not translate into higher ADG, as steers in this group exhibited a lower ADG of 707 g/day (*p* < 0.05). Previous research indicated that brassicas had no impact on dry matter intake (DMI) and ADG when compared to silage and hay [38]. Nevertheless, a reduction in DMI was observed in dairy cows and sheep fed brassicas [19,39]. Turnips are known for their high digestibility. As a result, the group with a high turnip content had a high dry matter intake. The Control diet exhibited a higher NDF content (43.1%) in contrast to the turnip diets, which had 34.9% for T50 and 33.8% for T70. This difference may result in increased intake when consuming turnip diets. The reduced ADG observed in the steers on turnip diets may be attributed to a brief period of adjustment to the new diet, as all the steers had been grazing on pasture before this study commenced (Appendix A). The fifteen-day period was likely inadequate for ruminal adaptation [40], as no weight gain was noted in the initial weeks when compared to the Control group, despite the high DMI. This is supported by [17], who recommends a careful adaptation period of at least five weeks when animals are fed sole diets of forage brassicas to allow for proper ruminal microbial adjustment and prevent performance setbacks.

The greater dorsal fat thickness observed in the Control group (*p* < 0.05) may be attributed to a more efficient utilization of metabolizable energy for fat deposition, whereas the increased gut fill associated with turnip supplementation may have reduced the availability of energy for subcutaneous fat accretion [20]. Furthermore, a longer adaptation period to the brassica-based diets might have improved nutrient digestibility and energy utilization [17]. Variations in steer growth rates, which were influenced by the dietary treatments, can account for the differences observed in fat thickness. The Control group exhibited greater daily live weight gain (1.4 kg vs. 0.9 kg), which likely contributed to the increased subcutaneous fat deposition. Thus, the type of diet indirectly affected fat thickness by modulating growth performance [41]. Steers from the Control group were processed sooner than those receiving the turnip treatments. This was due to the transition in diet from pasture to turnips for the animals in the T50 and T70 groups, which impacted their initial body weight and, in turn, influenced the fat content of their carcasses.

The initial live weight, hot carcass weight, dressing percentage, and muscle pH did not differ among treatments (*p* > 0.05). In this study, the average carcass weight was found to be lower compared to the national Chilean average (225 kg compared to 250 kg, respectively), as reported by Larraín and Vargas-Bello [42]. It was also lower than the average weight of carcasses from animals of the same breed that were finished using the traditional grazing system in Chile, which averaged 274 kg [3]. The meat pH fell within the normal range for bovine meat, which is from 5.4 to 5.9, and aligned with previously reported values [4,43,44].

### 3.2. Meat Quality

Meat quality characteristics from steers fed the different diets are presented in Table 4. No differences in instrumental L* values were observed for meat between treatments (*p* > 0.05). The meat from steers in the T50 and T70 treatment groups exhibited a redder hue (higher a*, *p* = 0.001) compared to the meat from steers on the Control diet. Additionally, the meat from steers fed the T70 diet showed a greater b* value (*p* = 0.006) in contrast to the meat from those on the Control diet, whereas the T50 group’s meat was intermediate and showed no significant difference from the other treatments. The increased redness levels in meat from the T50 and T70 groups may be due to different factors. Compared with the Control diet, turnip supplementation resulted in higher content of Fe in meat, which supports myoglobin synthesis, intensifying the red hue [45]. Another potential contributor to the improved meat redness in steers fed turnips could be the presence of tannins, which are known to possess antioxidant properties that may enhance meat color stability [46,47]. The b* value in meat is influenced by marbling, with higher marbling grades potentially leading to increased b* values [48]. Both the intramuscular fat (*p* = 0.009) and visual marbling (*p* < 0.001) were found to be higher in meat from steers in the T50 treatment groups compared to the Control group.

The L* values observed in meat from this study aligned closely with those documented by Duckett et al. [48], who examined meat from Angus-cross animals fed with various forages such as mixed pastures, alfalfa, and pearl millet. Similarly, the meat color values for a* and b* matched the findings of Morales et al. [4], who studied meat from comparable animals finished in the same region in Chile.

The a* values observed in this study were greater, whereas the b* values were less than those documented by Latimori et al. [49] for beef originating from Argentina’s Pampeana region. According to Holman et al. [50], a threshold a* value of 14.5 or higher is necessary for consumer acceptance of beef color. This suggests that the redness levels measured in the meat from this study would be appealing to consumers [51,52]. Carcasses from steers fed turnip diets exhibited lower lightness (L*) values in subcutaneous fat compared to the Control group (*p* < 0.05). This difference may be attributed to the thinner fat layer in turnip-fed carcasses, which could have allowed the colorimeter to detect the darker hue of the underlying muscle tissue. Additionally, although differences in pigment concentration or fat composition (e.g., melting point) might have influenced fat color, these factors were not evaluated in this study [51,53].

Shear force values were greater (*p* = 0.002) for meat from steers fed the Control diet, indicating tougher meat than treatments T50 and T70. The increased meat tenderness observed in the turnip-fed steers may be related to lower physical activity due to the readily available feed and possibly to muscle biochemical changes associated with the diet, including the modestly higher intramuscular fat content, rather than to an overall greater nutrient intake [52]. Shear force values from this study were similar to those reported by Hur et al. [53] for meat from Holstein steers (19.12 and 28.15 N) but inferior to those for beef from Hereford steers fed with pasture [51].

#### 3.2.1. Chemical Composition

The chemical composition of meat from the three treatments is shown in Table 5. Meat from the Control diet exhibited a significantly lower (*p* < 0.001) percentage of DM, crude protein, and total ash compared to meat from steers in the turnip treatment groups. The DM percentages observed in this study aligned with findings by Duckett et al. [48] and Leheska et al. [44], who reported 24–28% DM in beef. Supporting our findings, Leheska et al. [44] proposed that a higher fat content would lead to an increase in the percentage of DM in meat. The percentage of protein in muscle tissue shows less variation compared to the other components, and the results of this study, which ranged from 20.5% to 21.6%, align closely with the findings of other researchers, who reported a range of from 21% to 22.5% [37,54,55,56].

The percentage of intramuscular fat was notably greater (*p* = 0.009) in the meat from the T50 treatment compared to the meat from the Control group. Forage turnips are chemically composed of a high ratio of readily fermentable carbohydrates to structural carbohydrates (RFC:SC), which exceeds the ratio found in pastures like perennial ryegrass [17,57]. This swift fermentation of the carbohydrates in the rumen promotes the generation of propionate by bacteria [17,58]. Propionic acid, when combined with corn in the diet (which enhances starch digestion and releases glucose in the small intestine), is likely to boost the availability of glucose for muscles [57]. This would cause the intramuscular adipocytes to expand their fat storage as a result of increased de novo synthesis of fatty acids [59,60]. Additionally, the rise in blood glucose concentrations stimulates insulin secretion, which plays a key role in IMF deposition by enhancing glucose uptake in adipose tissue and activating lipogenic pathways [61,62].

According to Morales et al. [3], an average IMF content of 2.2% was identified in dairy and British cattle breeds fed solely on pasture or a combination of pasture and concentrate within the same production region as the current study. The lower IMF values observed in this study could be attributed to the lighter carcass weights compared to those reported by Morales et al. [3]. As noted by Pethick et al. [61], there is a correlation between carcass weight and intramuscular fat, with marbling increasing linearly as carcass weight ranges from 200 to 450 kg, a range where there is significant potential for fat deposition.

#### 3.2.2. Mineral Composition

Beef from steers fed turnips exhibited higher levels of sodium (*p* = 0.036) and manganese (*p* = 0.005) compared to beef from animals that consumed the Control diet. Additionally, there was a significant difference in iron content (*p* = 0.022), with meat from the T50 and T70 diets containing more iron than meat from the Control diet. Findings from the mineral composition analysis imply that adding forage turnip to animal diets could lead to a rise in sodium, iron, and manganese levels in meat. Nonetheless, the specific mechanism behind this is not well-documented in existing research. Notably, higher levels of these minerals have been observed in Holstein Friesian breeds compared to other breeds, which may be related to their increased metabolic activity [63].

### 3.3. Sensory Evaluation of Meat Quality

Results from sensory evaluation by a trained panel are shown in Table 6. There were differences between treatments in meat color intensity (*p* = 0.025) and degree of marbling of raw beef samples (*p* < 0.001). Color intensity was similar between Control and T50 samples but greater than in T70. The degree of marbling was elevated for T50 compared to both T70 and Control beef samples, which did not differ. Mean marbling values agree with IMF values from chemical analysis, with the highest fat percentage in T50 samples and the lowest percentage in Control samples. There were no differences in beef juiciness (*p* = 0.300), flavor (*p* = 0.558), or tenderness scores (*p* = 0.682) between cooked beef samples from the different dietary treatments. Meat quality at the time of consumption is influenced by various factors throughout the production chain, including the animal’s diet and its impact on meat composition. Various studies across different meat-producing species have demonstrated that higher intramuscular fat (IMF) levels in meat can enhance its eating quality [64,65,66]. Although the IMF percentage in meat from T50 was higher than in the Control group (1.79% vs. 1.12%), this study showed no noticeable differences in sensory evaluations. This may be explained by the fact that the IMF values in both groups remained below the threshold typically associated with perceptible improvements in sensory traits, which is reported to be around 3–4% in beef for untrained consumers [67]. Therefore, larger differences in IMF content may be necessary to produce detectable effects on sensory quality. Shear force measurements showed higher values for meat from the Control diet at 22.55 N, compared to 18.9 and 19.0 N for meat from steers that were fed T50 and T70, respectively. Despite this difference, trained panelists did not assign varying meat tenderness scores across dietary treatments.

The increased sensitivity of the instrument in identifying shear force differences in beef, as opposed to the panelists’ tenderness evaluations, could partially explain the differences in tenderness results between the instrumental and sensory methods. Most consumers consider these values acceptable, making them challenging for even a trained panel to discern. Holman et al. [68] suggested that a shear force value of less than 42.6 N is ideal for consumer satisfaction concerning beef tenderness. In contrast, Braña et al. [43] classified meat as very tender when the shear force values were below 31.3 N. The effects of feeding brassica on meat quality have been previously observed in lambs. Findings from the current study align with those of De Brito et al. [52], who noted no differences in the sensory qualities of meat from lambs fed various forages, including brassica. However, Hopkins et al. [69] observed that lambs fed brassica (*Brassica napus*) produced meat with a more pronounced flavor and aroma compared to those fed on perennial ryegrass.

### 3.4. Fatty Acids Composition and Cholesterol

Total lipid and FA compositions are shown in Table 7. Total fatty acid methyl esters in the *Longissimus thoracis et lumborum* muscle were highest (*p* = 0.011) in T50 compared with Control, whereas T70 did not differ (*p* > 0.05) from the other treatments.

The most representative SFAs were 16:0 and 18:0, irrespective of the diet, aligning with the values documented in other research [2,3,70].

Branched fatty acids (BCFAs), such as iso-15:0 and iso-17:0, differed among dietary treatments. Iso-15:0 was higher in the Control than the turnip treatments, whereas iso-17:0 was higher in the Control than T70, with T50 being intermediate, which did not differ from the other diets. BCFAs form an emerging group of bioactive fatty acids that are gaining growing interest in the scientific community due to their biological potential health benefits. Since BCFAs predominantly originate from bacteria located in the rumen, products from ruminant animals provide distinctive dietary sources of these fatty acids [71].

Including forage turnip in the diet resulted in a higher PUFA content in meat. Total PUFA levels were higher in T50 and T70 (*p* = 0.017), with T50 showing a notably greater *n*-6 content than the Control group, particularly due to an increase in 18:2 *n*-6 (*p* = 0.039). This could be attributed to the significant content of *n*-6 fatty acids in the T50 diet (Table 1). Similarly, when animals are fed corn, the levels of *n*-6 fatty acids rise compared to those in steers that consume forage [72].

Conversely, treatments involving the consumption of turnips showed an increase in *n*-3 fatty acids (*p* = 0.009), with levels of EPA and DPA being greater in the turnip treatment compared to the Control group (Table 7). This suggests that the 18:3 *n*-3 fatty acids present in the turnip treatments were digested and absorbed more efficiently, resulting in a greater incorporation into the intramuscular fat of the muscle tissue compared to the Control diet. A similar outcome was observed in lambs as documented by De Brito et al. [52]. Furthermore, secondary metabolites present in turnips might aid in safeguarding against the microbial biohydrogenation of PUFA, promoting their enhanced absorption and storage [73]. Additional explanations for the findings include the observation that feeding brassica has been associated with a rapid passage rate through the rumen [17,74], allowing a greater portion of consumed PUFA to bypass ruminal biohydrogenation and reach the duodenum unaltered. Another potential explanation is the elevated proportion of swiftly fermentable carbohydrates compared to structural carbohydrates in brassica. These are rapidly fermented in the rumen, reducing pH due to the increased production of volatile fatty acids [39]. This drop in ruminal pH impacts the biohydrogenation process of unsaturated fatty acids during the lipolysis phase, which results in an increased passage of these acids into the duodenum [75].

The ratios of PUFA:SFA and *n*-6:*n*-3 fatty acids remained consistent (*p* > 0.05) across different treatments. These ratios are commonly utilized to assess the nutritional quality of fats intended for human consumption. Foods are considered to have high nutritional value when they exhibit a PUFA:SFA ratio of 0.45 or above and an *n*-6:*n*-3 ratio below 4 [76]. In this study, the *n*-6:*n*-3 ratios were lower than 4 in all treatments, indicating a favorable balance of essential fatty acids. However, the PUFA:SFA ratios were below the recommended threshold of 0.45 across all treatments, with no significant differences observed among the dietary treatments (*p* = 0.098). Such low PUFA:SFA ratios have been associated with an increased risk of cardiovascular diseases, as SFAs are known to raise LDL cholesterol levels, whereas PUFAs contribute protective effects [77]. Despite this, ruminant meat remains a valuable source of essential nutrients, and dietary interventions aimed at increasing PUFA content could help enhance its health-promoting properties. The ratio of 11t-18:1 to 10t-18:1 was greater in the Control group compared to T50, with T70 showing an intermediate value. Levels of *trans*-vaccenic acid (TVA; 18:1 t11), a key intermediate in the ruminal biohydrogenation of unsaturated fatty acids and precursor of conjugated linoleic acid (c9, t11-18:2 CLA) [78], were numerically higher in the Control group. This finding aligns with reports that grass-based diets—rich in α-linolenic acid and with lower fermentability—enhance TVA formation due to incomplete biohydrogenation [79,80]. In contrast, diets based on rapidly fermentable carbohydrates like turnips may shift rumen fermentation patterns and microbial populations, favoring complete biohydrogenation and thereby reducing TVA accumulation [81]. In this context, beef from grass-fed animals has been reported to have higher levels of TVA [81]. The TVA levels found in this study were lower than those documented by Aldai et al. [82], Morales et al. [3], and Sales et al. [83]. No significant differences in CLA content were observed among the dietary treatments (*p* = 0.453).

Cholesterol levels were higher in the treatments containing forage turnips (T50: 34.6 mg/100 g fresh and T70: 33.6 mg/100 g fresh) compared to the Control (23.6 mg/100 g fresh) (*p* < 0.05). However, no significant differences in cholesterol content were observed between the forage turnip treatments. Duckett et al. [84] suggest that variations in cholesterol content are likely to be evident in meat samples that exhibit significantly different amounts of intramuscular fat, particularly those with extreme marbling scores. In this study, T50 showed a higher IMF% (Table 5), higher sum of FAME (Table 7), and higher visual marbling level (Table 6) than the Control, whereas T70 had intermediate total lipid levels, which did not differ from the other diets, except for visual marbling which was similar to T50 and higher than the Control [85].

## 4. Conclusions

Feeding forage turnips to dairy steers in their finishing phase resulted in reduced subcutaneous fat thickness (0.29 cm in T50 and 0.25 cm in T70 vs. 0.51 cm in the Control) and lower shear force (18.92 N in T50 and 19.02 N in T70 vs. 22.55 N in the Control) while enhancing meat redness. Additionally, feeding with turnips increased intramuscular fat (1.79% in T50 vs. 1.12% in the Control), polyunsaturated fatty acids, particularly *n*-3, and specific minerals, including iron, manganese, and sodium. However, the sensory qualities of the cooked beef remained unchanged compared to steers fed a conventional pasture and concentrate diet. Introducing 50% turnips in the diet is recommended as a viable alternative to pasture grazing during summer, ensuring a consistent nutrient supply for finishing steers and producing high-quality meat. However, increasing the inclusion level to 70% turnips did not provide further benefits in meat quality. Additionally, forage turnips can be integrated into pasture management strategies to enhance feed availability and quality during summer.

## Figures and Tables

**Table 1:** Ingredients and chemical composition (% of DM) of Control diet, 50% (T50) and 70% (T70) inclusion of forage turnips.

	Control ^1^	T50 ^1^	T70 ^1^
Pasture	68	-	-
Pasture hay	-	35	26
Rolled corn	-	15	4
Concentrate	32	-	-
Turnips	-	50	70
Chemical composition (%)			
Crude protein	14.7	14.3	16.5
Metabolizable energy (Mcal kg^−1^)	2.59	2.59	2.59
in vitro digestibility organic matter	78.2	80.4	82.9
Neutral detergent fiber	43.1	34.9	33.8
Acid detergent fiber	26.2	25.7	26.7
Ether extract	2.11	2.04	1.73
Ash	9.60	11.2	13.8
Carbohydrates	11.1	11.6	13.2
Σ FAMES ^2^ (mg100 g^−1^)	142.9	151.6	167.4
Fatty acids individuals (mg 100 g^−1^)			
16:0	21.1	26.0	30.8
18:0	2.77	2.16	3.04
c9–18:1	10.8	15.4	8.19
18:2 *n*-6	34.3	40.4	29.6
18:3 *n*-3	58.6	44.9	65.2
Σ SFA ^3^	29.4	32.6	38.6
Σ MUFA ^4^	17.7	30.7	30.0
Σ PUFA ^5^	93.2	85.5	95.1
Σ PUFA *n*-6	34.4	40.5	29.7
Σ PUFA *n*-3	58.8	45.1	65.3
Unknown ^6^	2.41	2.66	3.69

^1^ Control: pasture plus concentrate. T50: 50% turnips plus hay and corn. T70: 70% turnips plus hay and corn. ^2^ FAMES: Fatty acid methyl esters. ^3^ SFA: Saturated fatty acids. ^4^ MUFA: Monounsaturated fatty acids. ^5^ PUFA: Polyunsaturated fatty acids. ^6^ Unknown: Unidentified fatty acids.

**Table 2:** Botanical composition (%) of Control diet, 50% (T50) and 70% (T70) inclusion of forage turnips.

	Control ^1^	T50 ^1^/T70 ^1^
*Lolium perenne*	44.8 ± 20.9	-
*Dactylis glomerata*	11.9 ± 13.4	-
*Holcus lanatus*	3.8 ± 3.3	-
*Trifolium repens*	6.4 ± 5.1	-
*Turnips (Brassica rapa)*	-	97.8 ± 2.2
Others	8.2 ± 16.4	1.1 ± 0.4
Dead material	24.9 ± 9.5	1.1 ± 0.5

^1^ Control: pasture plus concentrate. T50: 50% turnips plus hay and corn. T70: 70% turnips plus hay and corn.

**Table 3:** Effect of animal feed type on performance and carcass traits of Holstein–Friesian steers (*n* = 9 by group).

	Control ^1^	T50 ^1^	T70 ^1^	*p*-Value	RMSE ^2^
Animal performance					
Initial live weight (kg)	391.4	390.2	388.9	0.936	14.842
Final live weight (kg)	449.7 ^a^	440.7 ^ab^	431.3 ^b^	0.037	14.194
DMI (kg DM/d) ^3^	8.69 ^b^	9.29 ^ab^	9.76 ^a^	0.009	1.953
ADG (kg/d) ^4^	0.972 ^a^	0.841 ^ab^	0.707 ^b^	0.008	0.138
Carcass traits					
pH	5.55	5.58	5.56	0.046	0.313
Fat thickness (cm)	0.51 ^a^	0.29 ^b^	0.25 ^b^	0.184	0.012
Hot carcass weight (kg)	228.1	226.2	221.8	6.621	0.135
Dressing (%)	50.7	51.3	51.5	1.513	0.151

^1^ Control: pasture plus concentrate; T50: 50% turnips plus hay and corn; T70: 70% turnips plus hay and corn. ^2^ RMSE, Root-mean-square error. ^3^ DMI: Dry Matter Intake. ^4^ ADG: Average Daily Gain. Letters within rows indicate means that significantly differ (*p* < 0.05).

**Table 4:** Effect of animal feed type on meat and fat color and shear force of the *Longissimus thoracis et lumborum* of Holstein–Friesian steers (*n* = 9 by group).

	Control ^1^	T50 ^1^	T70 ^1^	*p*-Value	RMSE ^2^
Meat color ^3^					
L*	40.6	38.8	40.2	0.251	2.29
a*	22.9 ^b^	25.9 ^a^	26.4 ^a^	0.001	1.74
b*	11.8 ^b^	13.0 ^ab^	13.7 ^a^	0.006	1.07
Fat color ^3^					
L*	67.0 ^a^	63.3 ^b^	63.5 ^b^	0.003	2.25
a*	9.72	10.9	12.1	0.154	2.5
b*	13.1	13.8	14	0.674	2.29
Shear force (N)	22.55 ^a^	18.92 ^b^	19.02 ^b^	0.002	2.15

^1^ Control: pasture plus concentrate; T50: 50% turnips plus hay and corn; T70: 70% turnips plus hay and corn. ^2^ RMSE, Root-mean-square error. ^3^ L*: lightness, a*: redness, b*: yellowness. Letters within rows indicate means that significantly differ (*p* < 0.05).

**Table 5:** Effect of animal feed type on the chemical composition of *Longissimus thoracis et lumborum* from steers (*n* = 9 by group).

	Control ^1^	T50 ^1^	T70 ^1^	*p*-Value	RMSE ^2^
Chemical composition (g 100 g^−1^)					
Dry matter	23.0 ^b^	24.8 ^a^	24.4 ^a^	<0.001	0.713
Crude Protein	20.5 ^b^	21.6 ^a^	21.5 ^a^	<0.001	0.519
Ash	0.99 ^b^	1.05 ^a^	1.05 ^a^	<0.001	0.036
IMF ^3^	1.12 ^b^	1.79 ^a^	1.60 ^ab^	0.009	0.421
Mineral composition					
Macro minerals (g 100 g^−1^)					
Phosphorus	0.80	0.80	0.79	0.911	0.076
Calcium	0.02	0.02	0.02	0.840	0.016
Magnesium	0.09	0.09	0.09	0.692	0.008
Sodium	0.21 ^b^	0.24 ^a^	0.24 ^a^	0.036	0.022
Potassium	1.48	1.44	1.44	0.722	0.139
Microminerals (mg 100 g^−1^)					
Zinc	10.40	10.96	11.26	0.253	1.088
Copper	0.754	0.893	0.844	0.338	0.197
Iron	5.14 ^b^	6.57 ^a^	6.22 ^ab^	0.022	1.044
Manganese	0.067 ^b^	0.159 ^a^	0.177 ^a^	0.005	0.068
Cholesterol (mg 100 g^−1^)	23.6 ^b^	34.6 ^a^	33.6 ^a^	0.021	8.561

^1^ Control: pasture plus concentrate; T50: 50% turnips plus hay and corn; T70: 70% turnips plus hay and corn. ^2^ RMSE, Root-mean-square error. ^3^ IMF: intramuscular fat; Letters within rows indicate means that significantly differ (*p* < 0.05).

**Table 6:** Effect of animal feed type on sensory characteristics assessed by a trained panel of raw and cooked *Longissimus thoracis et lumborum* from steers (*n* = 9 by group).

	Control ^1^	T50 ^1^	T70 ^1^	*p*-Value	RMSE ^2^
Raw beef samples					
Meat color	4.44 ^a^	4.53 ^a^	4.24 ^b^	0.025	0.92
Fat color	3.53	3.52	3.52	0.997	1.27
Marbling	2.39 ^b^	2.99 ^a^	2.65 ^b^	<0.001	1.04
Cooked beef samples					
Juiciness	4.69	4.39	4.60	0.300	1.70
Tenderness	6.56	6.41	6.52	0.682	1.60
Flavor	5.48	5.54	5.33	0.558	1.65

^1^ Control: pasture plus concentrate; T50: 50% turnips plus hay and corn; T70: 70% turnips plus hay and corn. ^2^ RMSE, Root-mean-square error. Letters within rows indicate means that significantly differ (*p* < 0.05).

**Table 7:** Effect of animal feed type on the fatty acid content (mg/100 g fresh meat) of *Longissimus thoracis et lumborum* from steers (*n* = 9 by group).

	Control ^1^	T50 ^1^	T70 ^1^	*p*-Value	RMSE ^2^
Σ FAME ^3^	1121.0 ^b^	1981.8 ^a^	1747.5 ^ab^	0.011	563.6
Σ SFA ^4^	664.2	636.9	642.8	0.572	46.27
10:0	0.96	0.45	0.53	0.068	0.384
12:0	1.12	0.88	0.71	0.058	0.306
13:0	0.48	0.57	0.48	0.359	0.138
14:0	33.5	34.2	32.5	0.788	5.236
15:0	4.89	4.31	4.55	0.348	0.683
16:0	379.7	386.5	385.9	0.867	24.23
17:0	13.2	12.2	13.0	0.406	1.472
18:0	226.7	194.7	201.9	0.195	30.87
19:0	1.44	1.44	1.40	0.962	0.392
20:0	1.30	1.02	1.15	0.252	0.289
22:0	0.76	0.57	0.69	0.146	0.162
Σ BCFA ^5^	22.94	19.88	20.68	0.241	3.142
iso-14:0	0.60	0.75	0.64	0.420	0.211
iso-15:0	2.62 ^a^	1.97 ^ab^	1.95 ^b^	0.034	0.476
iso-16:0	2.35	2.50	2.51	0.799	0.482
iso-17:0/6–8t-16:1	5.72 ^a^	4.77 ^b^	4.96 ^b^	0.013	0.532
iso-18:0	1.74	1.67	1.67	0.876	0.280
anteiso-15:0	2.35	1.67	1.94	0.098	0.523
anteiso-17:0/13t o 3t-16:1	7.56	6.54	7.02	0.347	1.191
Σ MUFA ^6^	745.6	729.8	732.8	0.806	43.97
Σ MUFA cis	683.8	672.5	676.0	0.903	44.28
c9-14:1	8.46	10.09	8.69	0.726	4.091
c7-16:1	3.75	3.36	3.39	0.369	0.519
c9-16:1	59.2	63.1	62.5	0.864	13.30
c11-16:1	1.69	2.20	1.83	0.414	0.713
c13-16:1	0.25	0.37	0.38	0.420	0.190
c9-17:1	10.6	11.1	12.0	0.178	1.406
c9-18:1	568.4	547.6	553.1	0.618	37.42
c11-18:1	22.3	25.4	25.3	0.277	3.721
c12-18:1	1.37	1.28	1.12	0.227	0.282
c13-18:1	4.29	4.84	4.34	0.664	1.249
c15-18:1	1.13	1.00	0.96	0.406	0.240
c9-19:1	0.24	0.21	0.28	0.318	0.090
c11–20:1	1.76	1.62	1.68	0.954	0.758
Σ MUFA *trans*	61.8	57.3	56.9	0.219	5.368
t9-16:1	0.94	1.08	0.91	0.156	0.177
t10-16:1	0.14	0.22	0.17	0.367	0.098
t11/t12-16:1	0.92	1.07	0.86	0.135	0.210
t6/t8-18:1	1.64 ^a^	1.25 ^ab^	1.2 ^b^	0.042	0.308
t9-18:1	2.50	2.30	2.29	0.210	0.228
t10-18:1	2.81	2.56	2.49	0.729	0.762
t11-18:1	17.2	12.7	13.3	0.063	3.371
t12-18:1	2.26	2.05	1.89	0.264	0.421
Σ PUFA ^7^	111.7 ^b^	141.4 ^a^	134.6 ^a^	0.017	16.97
Σ *n*-6	68.1 ^b^	86.6 ^a^	79.4 ^ab^	0.035	11.49
18:2 (*n*-6)	43.4 ^b^	55.3 ^a^	49.0 ^ab^	0.039	7.612
20:3 (*n*-6)	4.52	5.21	5.20	0.166	0.699
20:4 (*n*-6)	18.4	23.5	23.0	0.054	3.809
22:4 (*n*-6)	1.12	1.60	1.33	0.077	0.351
Σ *n*-3	41.3 ^b^	52.7 ^a^	52.9 ^a^	0.009	6.780
18:3 (*n*-3)	14.1	16.8	16.5	0.094	2.262
20:4 (*n*-3)	2.02	2.05	2.02	0.981	0.318
20:5 (*n*-3)/24:0	9.67 ^b^	12.9 ^a^	13.7 ^a^	0.008	2.197
22:5 (*n*-3)	13.7 ^b^	18.1 ^a^	18.0 ^a^	0.015	2.691
22:6 (*n*-3)	1.56	2.49	2.40	0.049	0.678
Σ CLA ^8^	5.98	5.15	5.27	0.453	1.22
c9, t11-18:2	5.48	4.71	4.77	0.448	1.15
t11-18:1/t10-18:1	6.53 ^a^	5.04 ^b^	5.64 ^ab^	0.025	1.08
*n*-6:*n*-3	1.66	1.66	1.50	0.098	0.18
PUFA: SFA	0.25	0.21	0.22	0.629	0.08
Cholesterol (mg 100 g^−1^)	23.6 ^b^	34.6 ^a^	33.6 ^a^	0.021	8.561

^1^ Control: pasture plus concentrate; T50: 50% turnips plus hay and corn; T70: 70% turnips plus hay and corn. ^2^ RMSE, Root-mean-square error. ^3^ FAME: Fatty acid methyl esters. ^4^ SFA: Saturated fatty acids. ^5^ BCFA: Branched-chain saturated fatty acid. ^6^ MUFA: Monounsaturated fatty acids. ^7^ PUFA: Polyunsaturated fatty acids. ^8^ CLA: Conjugated linoleic acid. Letters within rows indicate means that significantly differ (*p* < 0.05).

**Figure A1:**
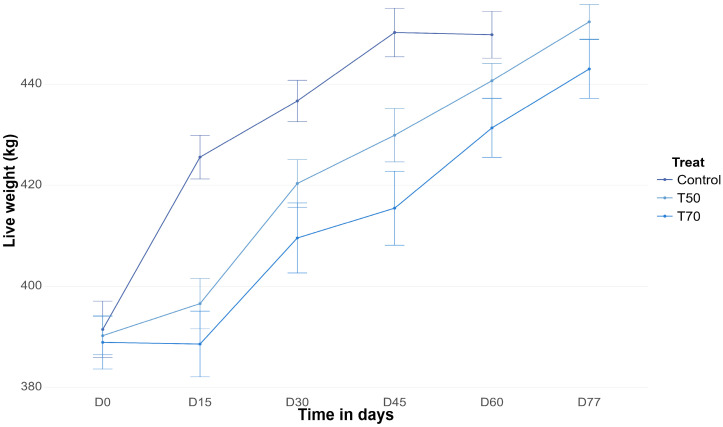
Fortnightly live weight of steers subjected to dietary treatments with 0% (Control), 50% (T50), and 70% (T70) forage turnip (*Brassica rapa* L.) inclusion during the finishing phase.

## Data Availability

The data that support the findings of this study are available from the corresponding author upon reasonable request.
